# Supplementary material for: Patient preferences in the treatment of hemophilia A: A latent class analysis
Source: PLoS One. 2021 Aug 23;16(8):e0256521. doi: 10.1371/journal.pone.0256521 (PMC8382185; doi:10.1371/journal.pone.0256521)

**Supplementary material for web-only publication (included for clarity of readers)**

**S1 Fig. Screenshot of the example choice set.** The question is: “You are diagnosed with hemophilia A. The doctor asks you to choose between therapy A, therapy B and therapy C. In your opinion, which therapy is the best and which is the worst?”


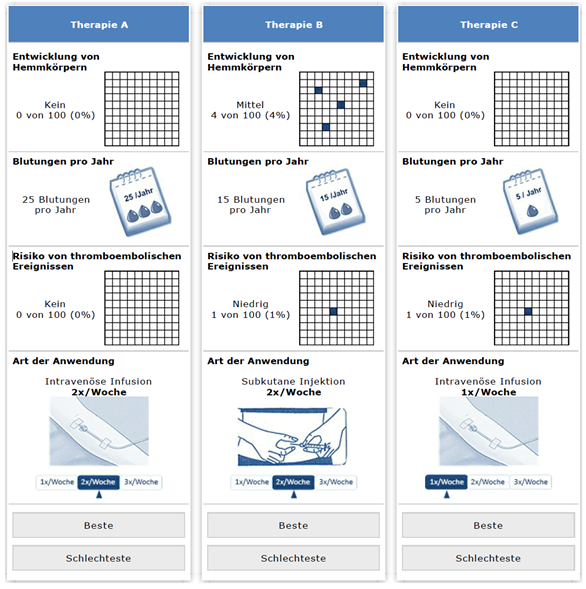

Supplement: S1 Fig — The question is: “You are diagnosed with hemophilia A. The doctor asks you to choose between therapy A, therapy B and therapy C. In your opinion, which therapy is the best and which is the worst?”. (DOCX) [file pone.0256521.s001.docx]
