# Supplementary material for: Patient preferences in the treatment of hemophilia A: A latent class analysis
Source: PLoS One. 2021 Aug 23;16(8):e0256521. doi: 10.1371/journal.pone.0256521 (PMC8382185; doi:10.1371/journal.pone.0256521)
Supplement: S2 Fig — Coefficients are displayed separately for each of the two classes. Vertical bars around the coefficients represent the 95% confidence interval. When the confidence intervals overlap for adjacent levels within an attribute, the coefficients of these levels are statistically not different from each other. The upper graphic shows coefficients of class 1 respondents, the lower graphic coefficients of class 2 respondents. The first attribute shows the two types of application, intravenous and subcutaneous, on top of each other. Also, the greater distance between the values “5 bleedings” and “15 bleedings” as well as between “15 bleedings” and “25 bleedings” compared with the distance between 0 and 5 bleedings of the attribute “Bleeding frequency per year” is taken into account in the graph. (DOCX) [file pone.0256521.s002.docx]

**Supplementary material for web-only publication (included for clarity of readers)**

**S2 Fig.** Coefficients of the Latent Class model.


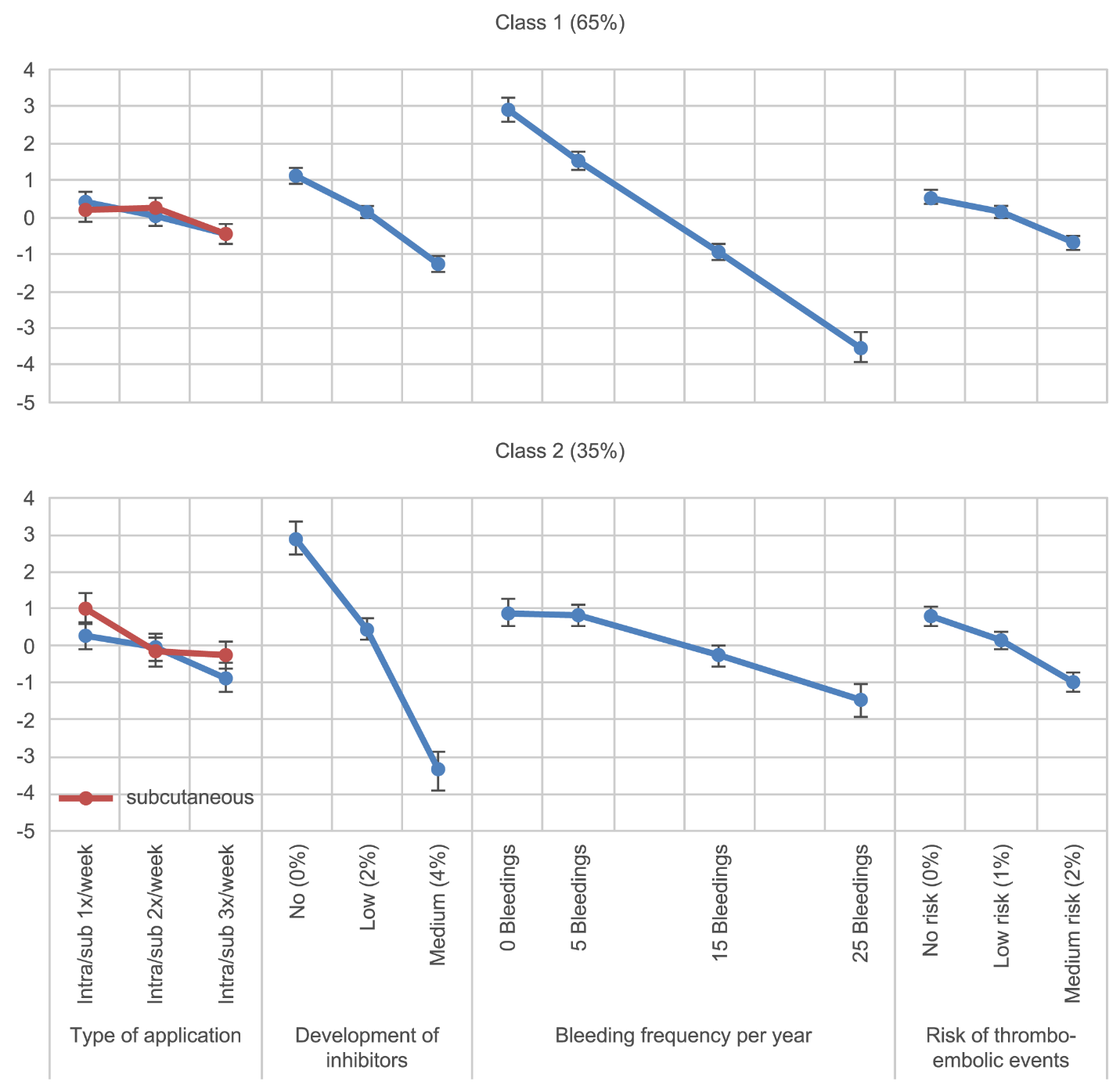
Coefficients are displayed separately for each of the two classes. Vertical bars around the coefficients represent the 95% confidence interval. When the confidence intervals overlap for adjacent levels within an attribute, the coefficients of these levels are statistically not different from each other. The upper graphic shows coefficients of class 1 respondents, the lower graphic coefficients of class 2 respondents. The first attribute shows the two types of application, intravenous and subcutaneous, on top of each other. Also, the greater distance between the values “5 bleedings” and “15 bleedings” as well as between “15 bleedings” and “25 bleedings” compared with the distance between 0 and 5 bleedings of the attribute “Bleeding frequency per year” is taken into account in the graph.
