# Supplementary material for: Patient preferences in the treatment of hemophilia A: A latent class analysis
Source: PLoS One. 2021 Aug 23;16(8):e0256521. doi: 10.1371/journal.pone.0256521 (PMC8382185; doi:10.1371/journal.pone.0256521)
Supplement: S1 Table — * P < 0.1, ** P < 0.05, *** P < 0.01. (DOCX) [file pone.0256521.s003.docx]

**Supplementary material for web-only publication (included for clarity of readers)**

**S1 Table. Characteristics of the therapy and experiences.**

**Bleedings**

|  | Class 1 (65%) | | Class 2 (35%) | |  |
| --- | --- | --- | --- | --- | --- |
| **Attribute** | ***N*** | ***%*** | ***N*** | ***%*** | **Chi 2 – Significance** |
| **How many bleedings have you had in the last year?** |  |  |  |  | (0.158) |
| 0 bleedings | 10 | *27.0* | 1 | *5.0* |  |
| 1–2 bleedings | 11 | *29.7* | 5 | *25.0* |  |
| 3–6 bleedings | 11 | *29.7* | 9 | *55.0* |  |
| >6 bleedings | 5 | *13.5* | 5 | *25.0* |  |
|  |  |  |  |  |  |
| **How many bleedings have you had in the last year?**  *(combined categories)* |  |  |  |  | * (0.054) |
| 0–2 bleedings | 21 | *56.8* | 6 | *30* |  |
| >2 bleedings | 16 | *43.2* | 14 | *70* |  |
|  |  |  |  |  |  |
| **Was there a period in your life when you had more bleeding than last year? If so, what was the maximum number of bleedings?** |  |  |  |  | * (0.088) |
| There was no such phase of life | 1 | *2.7* | 0 | *0.0* |  |
| 1–2 bleedings | 3 | *8.1* | 1 | *5.0* |  |
| 3–6 bleedings | 11 | *29.7* | 3 | *15.0* |  |
| 7–10 bleedings | 6 | *16.2* | 3 | *15.0* |  |
| 11–20 bleedings | 8 | *21.6* | 1 | *5.0* |  |
| >20 bleedings | 8 | *21.6* | 12 | *60.0* |  |
|  |  |  |  |  |  |
| **How many bleedings do you think a hemophilia A patient has on average per year?** |  |  |  |  | (0.906) |
| 0 bleedings | 0 | *0.0* | 0 | *0.0* |  |
| 1–2 bleedings | 1 | *2.7* | 1 | *5.0* |  |
| 3–6 bleedings | 19 | *51.4* | 10 | *50.0* |  |
| 7–10 bleedings | 5 | *13.5* | 2 | *10.0* |  |
| 11–20 bleedings | 9 | *24.3* | 4 | *20.0* |  |
| >20 bleedings | 3 | *8.1* | 3 | *15.0* |  |
|  |  |  |  |  |  |
| **Do you have "problem joints"? (Joints that cause you particularly severe or frequent problems/pains.)** |  |  |  |  | (0.316) |
| None | 9 | *24.3* | 10 | *50.0* |  |
| 1 joint | 7 | *18.9* | 3 | *15.0* |  |
| 2–3 joints | 16 | *43.2* | 4 | *20.0* |  |
| 4–5 joints | 3 | *8.1* | 2 | *10.0* |  |
| >5 joints | 2 | *5.4* | 1 | *5.0* |  |
|  |  |  |  |  |  |
| **How high was the proportion of joint bleeding last year (joint bleeding is defined as bleeding that occurs in the large joints such as knee, hip, elbow, ankle)?** |  |  |  |  | (0.264) |
| 0–25% | 22 | *59.5* | 13 | *65.0* |  |
| 26–50% | 2 | *5.4* | 3 | *15.0* |  |
| 51–75% | 3 | *8.1* | 1 | *5.0* |  |
| 76–100% | 10 | *27.0* | 2 | *10.0* |  |
| Not sure | 0 | *0.0* | 1 | *5.0* |  |

* P < 0.1

**Thromboembolic events**

|  | Class 1 (65%) | | Class 2 (35%) | |  |
| --- | --- | --- | --- | --- | --- |
| **Attribute** | ***N*** | ***%*** | ***N*** | ***%*** | **Chi 2 – Significance** |
| **What do you think is your risk of getting thrombosis or embolism?** |  |  |  |  | (0.576) |
| 0% | 14 | *37.8* | 8 | *40.0* |  |
| 1–2% | 19 | *51.4* | 9 | *45.0* |  |
| 3–5% | 4 | *10.8* | 2 | *10.0* |  |
| >5% | 0 | *0.0* | 1 | *5.0* |  |
|  |  |  |  |  |  |
| **How many thromboses or embolisms have you had in the past?** |  |  |  |  | (0.390) |
| 1 event | 1 | *2.7* | 2 | *10.0* |  |
| 2 events | 0 | *0.0* | 0 | *0.0* |  |
| 3 events | 1 | *2.7* | 0 | *0.0* |  |
| 4 events | 0 | *0.0* | 0 | *0.0* |  |
| 5 events | 0 | *0.0* | 0 | *0.0* |  |
| More than 5 events | 0 | *0.0* | 0 | *0.0* |  |
| No events | 35 | *93.8* | 18 | *90.0* |  |

**Development of inhibitors**

|  | Class 1 (65%) | | Class 2 (35%) | |  |
| --- | --- | --- | --- | --- | --- |
| **Attribute** | ***N*** | ***%*** | ***N*** | ***%*** | **Chi 2 – Significance** |
| **Have you ever had any inhibitors?** |  |  |  |  | (0.301) |
| Inhibitors present | 12 | *32.4* | 4 | *20.0* |  |
| No inhibitors | 23 | *62.2* | 16 | *80.0* |  |
| Not sure | 2 | *5.4* | 0 | *0.0* |  |
|  |  |  |  |  |  |
| **Have you ever taken a test to see if you were at risk for inhibitors?** |  |  |  |  | (0.562) |
| Performed | 33 | *89.2* | 17 | *85.0* |  |
| Not performed | 1 | *2.7* | 0 | *0.0* |  |
| Not sure | 3 | *8.1* | 3 | *15.0* |  |
|  |  |  |  |  |  |
| **What do you think: What is your risk of developing inhibitors?** |  |  |  |  | (0.598) |
| 0% | 14 | *37.8* | 6 | *30.0* |  |
| 2% | 17 | *45.9* | 8 | *40.0* |  |
| 4% | 2 | *5.4* | 3 | *15.0* |  |
| >4% | 4 | *10.8* | 3 | *15.0* |  |

**Type of application**

|  | Class 1 (65%) | | Class 2 (35%) | |  |
| --- | --- | --- | --- | --- | --- |
| **Attribute** | ***N*** | ***%*** | ***N*** | ***%*** | **Chi 2 – Significance** |
| **How often do you have to administer the drug in your current therapy?** |  |  |  |  | (0.269) |
| 1 x per week | 2 | *5.4* | 4 | *20.0* |  |
| 2–4 x per week | 26 | *70.2* | 8 | *40.0* |  |
| More than 4 x per week | 0 | *0.0* | 0 | *0.0* |  |
| Daily | 2 | *3.1* | 1 | *5.0* |  |
| On demand | 7 | *21.9* | 7 | *35.0* |  |
| Do not know | 0 | *0.0* | 0 | *0.0* |  |
|  |  |  |  |  |  |
| **What do you think: On average, how often does a hemophilia A patient have to inject the drug?** |  |  |  |  | (0.576) |
| 1 x per week | 3 | *8.1* | 1 | *5.0* |  |
| 2–4 x per week | 33 | *89.2* | 17 | *85.0* |  |
| More than 4 x per week | 0 | *0.0* | 0 | *0.0* |  |
| Daily | 0 | *0.0* | 1 | *5.0* |  |
| On demand | 0 | *0.0* | 1 | *5.0* |  |
| Do not know | 1 | *2.7* | 0 | *0.0* |  |
|  |  |  |  |  |  |
| **What do you think: Does subcutaneous administration of the drug have the same efficacy as intravenous infusion?** |  |  |  |  | (0.527) |
| Yes | 10 | *27.0* | 3 | *15.0* |  |
| No | 14 | *37.8* | 10 | *50.0* |  |
| Do not know | 13 | *35.1* | 7 | *30.0* |  |
|  |  |  |  |  |  |
| **Have you ever injected a drug subcutaneously?** |  |  |  |  | (0.599) |
| Yes | 14 | *37.8* | 9 | *45.0* |  |
| No | 23 | *62.2* | 11 | *55.0* |  |
| Do not know | 0 | *0.0* | 0 | *0.0* |  |
|  |  |  |  |  |  |
| **How is your hemophilia A currently treated?** |  |  |  |  | * (0.085) |
| Regular drug administration (prophylactic treatment) | 30 | *81.1* | 12 | *60.0* |  |
| Taking drugs as needed (on-demand treatment, substitution on demand) | 7 | *18.9* | 8 | *40.0* |  |
|  |  |  |  |  |  |
| **Does your treatment plan provide a subcutaneous or intravenous administration?** |  |  |  |  |  |
| Subcutaneous | 3 | *8.1* | 4 | *20.0* | (0.192) |
| Intravenous | 34 | *91.9* | 16 | *80.0* |  |

* P < 0.1

**Severity of hemophilia A**

|  | Class 1 (65%) | | Class 2 (35%) | |  |
| --- | --- | --- | --- | --- | --- |
| **Attribute** | ***N*** | ***%*** | ***N*** | ***%*** | **Chi 2 – Significance** |
| **What is the severity of your hemophilia A?** |  |  |  |  | *** (0.006) |
| Mild form (5%–40% of normal Factor VIII activity) | 5 | *13.5* | 0 | *0.0* |  |
| Moderate form (1%–5% of normal Factor VIII activity) | 3 | *8.1* | 8 | *40.0* |  |
| Severe form (less than 1% of normal Factor VIII activity) | 29 | *78.4* | 12 | *60.0* |  |
| Do not know | 0 | *0.0* | 0 | *0.0* |  |

*** P < 0.01

**Current state of health**

|  | Class 1 (65%) | | Class 2 (35%) | |  |
| --- | --- | --- | --- | --- | --- |
| **Attribute** | ***N*** | ***%*** | ***N*** | ***%*** | **Chi 2 – Significance** |
| **How would you describe your current state of health in general?** |  |  |  |  | ** (0.017) |
| Very good | 7 | *18.9* | 10 | *50.0* |  |
| Good | 23 | *62.2* | 4 | *20.0* |  |
| Satisfactory | 6 | *16.2* | 5 | *25.0* |  |
| Less good | 0 | *0.0* | 1 | *5.0* |  |
| Bad | 1 | *2.7* | 0 | *0.0* |  |

** P < 0.05
