# Supplementary material for: Patient preferences in the treatment of hemophilia A: A latent class analysis
Source: PLoS One. 2021 Aug 23;16(8):e0256521. doi: 10.1371/journal.pone.0256521 (PMC8382185; doi:10.1371/journal.pone.0256521)
Supplement: S2 Table — (DOCX) [file pone.0256521.s004.docx]

**Supplementary material for web-only publication (included for clarity of readers)**

**S2 Table.** **Sociodemographic characteristics of the overall sample and the two Latent Classes.**

|  | Class 1  (65%) | | | | Class 2  (35%) | | |
| --- | --- | --- | --- | --- | --- | --- | --- |
| **Characteristic** | ***N*** | | ***%*** | | ***N*** | | ***%*** |
| *Gender* |  |  | |  | |  | |
| Male | 35 | | *94.6* | | 19 | | *95.0* |
| Female | 2 | | *5.4* | | 1 | | *5.0* |
| *Age* |  | |  | |  | |  |
| 18–29 years | 17 | | *45.9* | | 10 | | *50.0* |
| 30–39 years | 6 | | *16.2* | | 3 | | *15.0* |
| 40–49 years | 7 | | *18.9* | | 4 | | *20.0* |
| 50–59 years | 4 | | *10.8* | | 3 | | *15.0* |
| 60–69 years | 3 | | *8.1* | | 0 | | *0.0* |
| Mean/standard deviation | 35.5/14.3 | | | | 33.2/12.2 | | |
| *Highest level of education* |  | |  | |  | |  |
| Junior/middle school certificate (8 classes) | 4 | | *10.8* | | 0 | | *0.0* |
| Intermediate high school, secondary school certificate (10 classes) | 10 | | *27.0* | | 5 | | *25.0* |
| Vocational school/advanced technical certificate | 2 | | *5.4* | | 0 | | *0.0* |
| Abitur high school diploma in Germany: university entrance qualification | 11 | | *29.7* | | 7 | | *35.0* |
| Technical college degree | 1 | | *2.7* | | 1 | | *5.0* |
| University degree | 8 | | *21.6* | | 6 | | *30.0* |
| Doctoral degree, PhD | 1 | | *2.7* | | 1 | | *5.0* |
| *Residential status* |  | |  | |  | |  |
| Spouse or partner | 20 | | *54.1* | | 8 | | *40.0* |
| One or more adult child (18 years or older) | 8 | | *21.6* | | 1 | | *5.0* |
| One or more minor child (under 18 years) | 10 | | *27.0* | | 7 | | *35.0* |
| One or both of their parents | 7 | | *18.9* | | 5 | | *25.0* |
| Alone | 7 | | *18.9* | | 6 | | *30.0* |
| *Employment status* |  | |  | |  | |  |
| Employed, full-time (>30 working hours per week) | 20 | | *54.1* | | 12 | | *60.0* |
| Employed, part-time (≤30 working hours per week) | 1 | | *2.7* | | 1 | | *5.0* |
| Self-employed/Freelance | 3 | | *8.1* | | 0 | | *0.0* |
| Student | 9 | | *24.3* | | 6 | | *30.0* |
| Retired or pensioner | 4 | | *10.8* | | 1 | | *5.0* |
