# Supplementary material for: Patient preferences in the treatment of hemophilia A: A latent class analysis
Source: PLoS One. 2021 Aug 23;16(8):e0256521. doi: 10.1371/journal.pone.0256521 (PMC8382185; doi:10.1371/journal.pone.0256521)
Supplement: S3 Table — CI = confidence interval, Coef. = coefficient, LCM = latent class model, SE = standard error, Sig = significance. *** P < 0.01, ** P < 0.05, * P < 0.1. ll(model): -709.411; AIC: 1474.821; BIC: 1651.773; degrees of freedom: 28. In the LCM analysis additional covariates were included as class-membership effects for the two classes in the model. Variables that showed a significant or an almost-significant level in the cross tables (see S1 Table) were tested as covariates in the latent class model. These variables were binary and assumed to be constant across alternatives for the same respondent. Only three covariates which showed a significance of at least P < 0.05 were included in the final model. The first covariate (cov1) was derived from the question “How many bleedings have you had in the last year?” and coded 1 if respondents had 0 to 2 bleedings last year and 0 otherwise. Second covariate (cov2) was derived from the question “What was the maximum number of bleedings?” The variable was coded 1 if respondents stated that they suffered from a maximum number of bleedings of more than 20 and 0 otherwise in the dataset. The third covariate (cov3) based on the question “How would you describe your current state of health in general?” It was coded 1 if respondents answered with very good and coded with 0 otherwise. Reference class is class 2. Respondents in class 1 differed significantly in terms of bleeding frequencies, maximum number of bleedings, and current state of health. Class 1 respondents had a lower number of bleedings in the last year (coef. = 2.04; P < 0.05). Respondents of class 2 were more likely to have a maximum number of bleedings of more than 20 (coef. = -2.56; P < 0.01). Regarding current state of health, class 1 had a significant lower proportion of respondents with a very good health state (self-report by respondents) (coef. = -2.91; P < 0.01) than class 2 respondents. (DOCX) [file pone.0256521.s005.docx]

**Supplementary material for web-only publication (included for clarity of readers)**

**S3 Table. Latent Class Model with membership variables.**

|  | Class 1 (68%) | | | | |  | | Class 2 (32%) | | | |
| --- | --- | --- | --- | --- | --- | --- | --- | --- | --- | --- | --- |
| **Level** | **Coef.** | **SE** | **95% CI** | | **Sig** |  | **Coef.** | **SE** | **95% CI** | | **Sig** |
| *Type of application* | | | | | | | | | | | |
| Intravenous 1x/week | 0.47 | 0.13 | 0.21 | 0.73 | *** |  | 0.22 | 0.20 | -0.17 | 0.62 |  |
| Intravenous 2x/week | 0.04 | 0.12 | -0.20 | 0.27 |  |  | 0.01 | 0.21 | -0.40 | 0.41 |  |
| Intravenous 3x/week | -0.51 | 0.14 | -0.78 | -0.25 | *** |  | -0.87 | 0.22 | -1.30 | -0.43 | *** |
| Subcutaneous 1x/week | 0.26 | 0.14 | -0.01 | 0.54 | * |  | 1.00 | 0.22 | 0.57 | 1.44 | *** |
| Subcutaneous 2x/week | 0.24 | 0.12 | 0.01 | 0.48 | ** |  | -0.18 | 0.22 | -0.60 | 0.24 |  |
| Subcutaneous 3x/week | -0.50 | 0.14 | -0.76 | -0.23 | *** |  | -0.19 | 0.20 | -0.59 | 0.21 |  |
| *Development of inhibitors* | | | | | | | | | | | |
| No (0%) | 1.12 | 0.10 | 0.93 | 1.31 | *** |  | 3.15 | 0.26 | 2.65 | 3.65 | *** |
| Low (2%) | 0.12 | 0.08 | -0.03 | 0.28 |  |  | 0.58 | 0.16 | 0.27 | 0.89 | *** |
| Medium (4%) | -1.25 | 0.10 | -1.44 | -1.05 | *** |  | -3.73 | 0.32 | -4.35 | -3.10 | *** |
| *Bleeding frequency per year* | | | | | | | | | | | |
| 0 Bleedings | 2.79 | 0.15 | 2.49 | 3.09 | *** |  | 0.95 | 0.20 | 0.56 | 1.33 | *** |
| 5 Bleedings | 1.52 | 0.12 | 1.29 | 1.75 | *** |  | 0.84 | 0.16 | 0.53 | 1.16 | *** |
| 15 Bleedings | -0.92 | 0.11 | -1.14 | -0.71 | *** |  | -0.29 | 0.16 | -0.60 | 0.03 | * |
| 25 Bleedings | -3.38 | 0.19 | -3.75 | -3.01 | *** |  | -1.51 | 0.23 | -1.96 | -1.06 | *** |
| *Risk of thromboembolic events* | | | | | | | | | | | |
| No risk (0%) | 0.55 | 0.09 | 0.38 | 0.73 | *** |  | 0.90 | 0.15 | 0.61 | 1.18 | *** |
| Low risk (1%) | 0.13 | 0.08 | -0.03 | 0.29 |  |  | 0.13 | 0.12 | -0.11 | 0.37 |  |
| Medium risk (2%) | -0.68 | 0.09 | -0.85 | -0.51 | *** |  | -1.03 | 0.15 | -1.32 | -0.74 | *** |
| *Membership variables* |  |  |  |  |  |  |  |  |  |  |  |
| cov1 (0–2 bleedings last year) | 2.04 | 0.87 | 0.34 | 3.74 | ** |  |  |  |  |  |  |
| cov2 (max no. of bleedings >20) | -2.56 | 0.91 | -4.34 | -0.79 | *** |  |  |  |  |  |  |
| cov3 (very good health state) | -2.91 | 0.97 | -4.80 | -1.01 | *** |  |  |  |  |  |  |
| _cons | 2.10 | 0.78 | 0.56 | 3.63 | *** |  |  |  |  |  |  |
| CI = confidence interval, Coef. = coefficient, LCM = Latent Class model, SE = standard error, Sig = significance. *** P < 0.01,  ** P < 0.05, * P < 0.1. ll(model): -709.411; AIC: 1474.821; BIC: 1651.773; degrees of freedom: 28 | | | | | | | | | | | |

In the LCM analysis additional covariates were included as class-membership effects for the two classes in the model. Variables that showed a significant or an almost-significant level in the cross tables (see S2 Table) were tested as covariates in the Latent Class model. These variables were binary and assumed to be constant across alternatives for the same respondent. Only three covariates which showed a significance of at least P < 0.05 were included in the final model. The first covariate (cov1) was derived from the question “How many bleedings have you had in the last year?” and coded 1 if respondents had 0 to 2 bleedings last year and 0 otherwise. Second covariate (cov2) was derived from the question “What was the maximum number of bleedings?” The variable was coded 1 if respondents stated that they suffered from a maximum number of bleedings of more than 20 and 0 otherwise in the dataset. The third covariate (cov3) based on the question “How would you describe your current state of health in general?” It was coded 1 if respondents answered with very good and coded with 0 otherwise. Reference class is class 2. Respondents in class 1 differed significantly in terms of bleeding frequencies, maximum number of bleedings, and current state of health. Class 1 respondents had a lower number of bleedings in the last year (coef. = 2.04; P < 0.05). Respondents of class 2 were more likely to have a maximum number of bleedings of more than 20 (coef. = -2.56; P < 0.01). Regarding current state of health, class 1 had a significant lower proportion of respondents with a very good health state (self-report by respondents) (coef. = -2.91; P < 0.01) than class 2 respondents.
