# Supplementary material for: Patient preferences in the treatment of hemophilia A: A latent class analysis
Source: PLoS One. 2021 Aug 23;16(8):e0256521. doi: 10.1371/journal.pone.0256521 (PMC8382185; doi:10.1371/journal.pone.0256521)
Supplement: Attachment — Submitted filename: Pat_Preferences_Hemophilia_A_LClass_journal_response_210604.docx [file pone.0256521.s006.docx]

Axel C. Mühlbacher

Gesundheitsökonomie und Medizinmanagement

Hochschule Neubrandenburg

Brodaer Straße 2

17033 Neubrandenburg, Germany

07 June 2021

Dr. Katalin Lorincz

*PLOS ONE*

**Patient preferences in the treatment of hemophilia A: A Latent Class analysis
[PONE-D-20-34726R1]**

Dear Dr. Lorincz,

On behalf of my co-authors, thank you for providing us with the opportunity to further revise our manuscript for *PLOS ONE*. As requested, please find our responses to each query below.

Thank you once again for your time; I hope to hear from you in due course.

Kind regards,

Axel C. Mühlbacher

**Patient preferences in the treatment of hemophilia A: A Latent Class analysis
*PLOS ONE*
Ref.: PONE-D-20-34726R1**

**Response to editorial comments (due 23 June 2021)**

| **Comments** | **Response/change** |
| --- | --- |
| *I.) Thank you for including your ethics statement on the online submission form:*  *"Participants were recruited by an external market research company. Prior to participation, patients gave informed consent. Participation was voluntary and anonymous. Mühlbacher AC, Sadler A, Lamprecht B, Juhnke C. Patient Preferences in the Treatment of Hemophilia A: A Best–Worst Scaling Case 3 Analysis. Value Health. July 20 2020; in press. All documents used in the study were primarily reviewed by the ethics committee at the State Medical Association of Baden-Württemberg. The study was declared harmless and approved (F-2017-048). In addition, the study was notified to the State Medical Associations of Hessen, Lower Saxony and Saarland, as these are the chambers responsible for the study centers where patient recruitment took place. This study used an anonymous online data collection. All patients gave a virtual declaration of consent before the actual survey by clicking a correspoding box and thus agreed to participate in the research project. All participants were comprehensively informed and enlightened about the research project. The patients were free to answer the questions addressed to them or to terminate the questionnaire at any time during the survey. No IP addresses of the computers, tablets etc. used or other security relevant information was collected.*  To help ensure that the wording of your manuscript is suitable for publication, would you please also add this statement at the beginning of the Methods section of your manuscript file. | We have added the statement as requested on page 3–4 and removed the subsequent duplication of information from page 6. |
| *II.) Before we proceed, would you please kindly clarify the following points:*  1) Did the authors present any new data in this submission that were not previously presented in the published article: [https://www.valueinhealthjournal.com/article/S1098- 3015(20)30179-0/abstract]? | Unless reference 7 is cited, we confirm that the data are new.  The underlying methods for the study as a whole, as well as the overall sample population results were reported in the previous paper on best–worst scaling. The latent class model methods and results are exclusive to the current paper. |
| 2) Did the authors perform any additional experiments or collect any additional data that were not a part of the study from the published article: [https://www.valueinhealthjournal.com/article/S1098- 3015(20)30179-0/abstract]? | As above, the two papers used data from the same study. All of the latent class analyses are exclusive to the current paper and not reported in the previous paper on best–worst scaling. |
| *III.) Thank you for updating your Data Availability Statement and including the following information:  "The datasets generated and/or analyzed during the current study are not publicly available due to EU General Data Protection Regulation. Data are available for sharing after reasonable request and conforming to the use in health preference research (HPR) for noncommercial aims...Request to access study data should be sent to:* [*grenzach.biometrics-hub-europe-africa@roche.com*](mailto:grenzach.biometrics-hub-europe-africa@roche.com)*"  We note that you've included the following information in your Response to the Reviewers' Comments:  "We have added that the datasets generated and/or analyzed during the current study are not publicly available due to EU General Data Protection Regulation, but are available from the corresponding author on reasonable request..."  In line with our goal of ensuring long-term data availability to all interested researchers, PLOS’ Data Policy states that authors cannot be responsible for ensuring data access. Therefore, before we continue, please clarify the following:*  a) Please confirm whether the email you provided, "[grenzach.biometrics-hub-europe-africa@roche.com,](mailto:grenzach.biometrics-hub-europe-africa@roche.com,)" belongs to any of the authors of this submission. | We confirm that [grenzach.biometrics-hub-europe-africa@roche.com](mailto:grenzach.biometrics-hub-europe-africa@roche.com) does not belong to any of the authors on this submission. |
| b) If this is an author related email, please provide a non-author point of contact (e.g., an email address, a URL, etc.) where researchers can request access to your data.  We will update your Data Availability statement on your behalf to reflect the information you provide. | Please see above. Many thanks for updating the manuscript on our behalf. |
